# Novel perspectives on swept-source optical coherence tomography

**DOI:** 10.1186/s40942-016-0050-y

**Published:** 2016-11-01

**Authors:** Fabio Lavinsky, Daniel Lavinsky

**Affiliations:** 1 Department of Ophthalmology, Paulista School of Medicine, São Paulo Hospital, Federal University of São Paulo, São Paulo, Brazil; 2 Department of Ophthalmology, Federal University of Rio Grande do Sul, Porto Alegre, Brazil; 3 Department of Ophthalmology, Instituto de Oftalmologia Lavinsky, Rua Quintino Bocaiuva 673, Porto Alegre, RS 90410-140 Brazil

## Abstract

Technologies for multimodal digital imaging of vitreoretinal diseases have improved the accuracy of diagnosis and the depth of the knowledge of the mechanisms of disease and their response to treatments. Optic coherence tomography (OCT) has become a mandatory tool for the management and for the follow-up of retinal pathologies. OCT technology evolved in the last two decades from time-domain to spectral domain and recently to the swept-source OCTs (SS-OCT). SS-OCT improved the depth of imaging and the scan speed, thus adding novel algorithms and features such as for vitreous and vitreoretinal interface evaluation, choroid segmentation and mapping, OCT angiography and En-face OCT. The multimodal approach using SS-OCT is expected to advance the understanding of retinal pathologies such as age related macular degeneration, diabetic maculopathy, central serous chorioretinopathy, the pachychoroid spectrum and macular telangiectasia. Surgical vitreoretinal diseases such as vitreo-macular traction syndrome, epiretinal membrane, retinal detachment, proliferative vitreoretinal retinopathy and diabetic traction retinal detachment also will be better understood and documented with SS-OCT. This technology also provides great utility for a broad spectrum of ophthalmic pathologies including glaucoma, uveitis, tumors and anterior segment evaluation.

## Introduction

Optic coherence tomography (OCT) is a technology introduced by Huang and associates [1] that became the prevailing technology for noninvasive assessment of the anterior and posterior segments.

The OCT is an imagining modality that enables the documentation of tissue structure or pathologies in real time and in situ with a resolution from 1 to 15 µm [2]. The eye is the most optically accessible organ of the human body, both anterior and posterior segments can be documented [3]. OCT can be used to measure different structures of the retina and is useful for the diagnosis and for the detection of progression of conditions such as macular edema and glaucoma [4, 5].

Time-domain OCT (TD-OCT) uses low-coherence light that split into two beams at a partially reflecting mirror, one is aimed at the tissue and the other at the moving reference arm. The beams then recombine at a photodetector and the interference is assessed to determine the measurement of the tissues [2]. Spectral-domain OCT (SD-OCT) was first demonstrated in 2002 [6, 7]. SD-OCT improved image resolution and scan speed [8]. Speed ranging from 29,000 to 80,000 scans per second and an axial resolution up to 2 µm were demonstrated [9, 10]. The increased number of images acquired improves the area of the retina evaluated, as well as the visualization of retinal structures. The higher speed of the SD-OCT acquisition enables it to obtain tridimensional volumetric data to show scans perpendicular to the scanning axis: C-scans. [11].

**Fig. 1:**
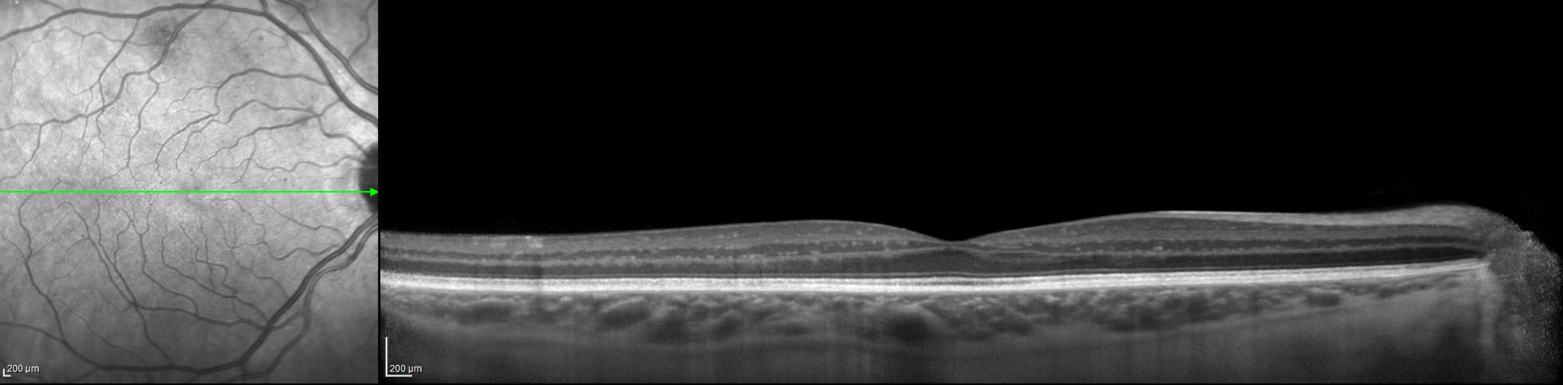
Spectral domain OCT with enhanced depth imaging (EDI) mode

Other features present in some commercially available SD-OCTs are the ART (*automatic real time*) mode that improves the resolution with noise reduction and the *eye*-*tracking* that follows the saccadic movements and can be used in combination with the ART mode [12]. The enhanced depth imaging (EDI) mode was developed to improve the visualization of deep structures such as external retina, retinal pigmentary epithelium (RPE), choroid and sclera [13] (Fig. 1).

The recently introduced SS-OCT uses a short cavity swept laser with a tunable wavelength of operation instead of the diode laser used in spectral-domain OCT [14]. The SS-OCT has improved image penetration using a wavelength of 1050 nm and has an axial resolution of 5.3 µm and a axial scan rate of 100.000 scans per second. Prototype models could reach faster scan-speeds of more than 400.000 scans per second [15, 16]. The 12 × 9 mm scan enables simultaneous imaging of the macula, the peripapillary area and the optic nerve head and the choroidal thickness. The 12 × 9 mm scan comprises 256 B-scans each comprising 512 A-scans with a total acquisition time of 1.3 s [17]. SS-OCT also provides the capability of a wide field up to 12 × 12 mm images [18]. SS-OCT enables clear simultaneous visualization of the vitreous and the posterior pre-cortical vitreous pockets and the choroid and the sclera [19].

The present review highlights some important advances in retinal diagnosis provided by SS-OCT. The SS-OCT commercially available in several countries, including Brazil, is the Triton^tm^ SS-OCT (Topcon, Tokyo, Japan). Its central wavelength is 1050 × nm and the speed scan is 100,000 A-scans per second.

There are other SS-OCTs that are still prototypes and to this date are not commercially available such as the Plex Elite Zeiss 9000, the vertical cavity surface emitting laser (VCSEL) OCT angiography from Massachusetts Institute of Technology (MIT) and a swept source megahertz OCT that operates with a 1050 nm wavelength and reaches scan speeds of 1.68 MHz [16]. The VCSEL ultra-high speed prototype operates at 1060 nm wavelength which allows increased light penetration into pigmented tissues and improved choroidal blood flow visualization. It obtains 500 × 500 A-scans at 400,000 A-scans per second in approximately 3.8 s. This ultra-high speed allows wider fields of view up to 12 × 12 mm. [18].

The scope of this review will include vitreous evaluation using the Enhanced Vitreous Visualization^tm^ (EVV), the choroid evaluation, En-face SS-OCT and OCT angiography (OCTA) and selected features of the SS-OCT utility in age-related macular degeneration (AMD), in central serous chorioretinopathy (CSCR), in diabetic maculopathy and in macular telangiectasia (Mactel).

## Vitreous structures imaging

The vitreous is a transparent hydrophilic gel, mainly composed of water, occupying the space between the lens and the retina and functions as a pathway for nutrients utilized by the lens, ciliary body and retina [20]. The advent of the spectral-domain OCT (SD-OCT) allowed better visualization of the vitreoretinal interface and posterior vitreous cortex and is widely used in the diagnosis and management of many diseases including: vitreomacualar traction (VMT), epiretinal membrane (ERM) [21], lamellar holes, pseudoholes and full thickness macular holes [20, 22]. However, swept-source OCT (SS-OCT) has enabled higher speed, longer imaging range and lower sensitivity roll-off compared with SD-OCT. SS-OCT uses a longer wave length and has less variation in sensitivity with depth (roll-off) compared with SD-OCT [15].

**Fig. 2:**
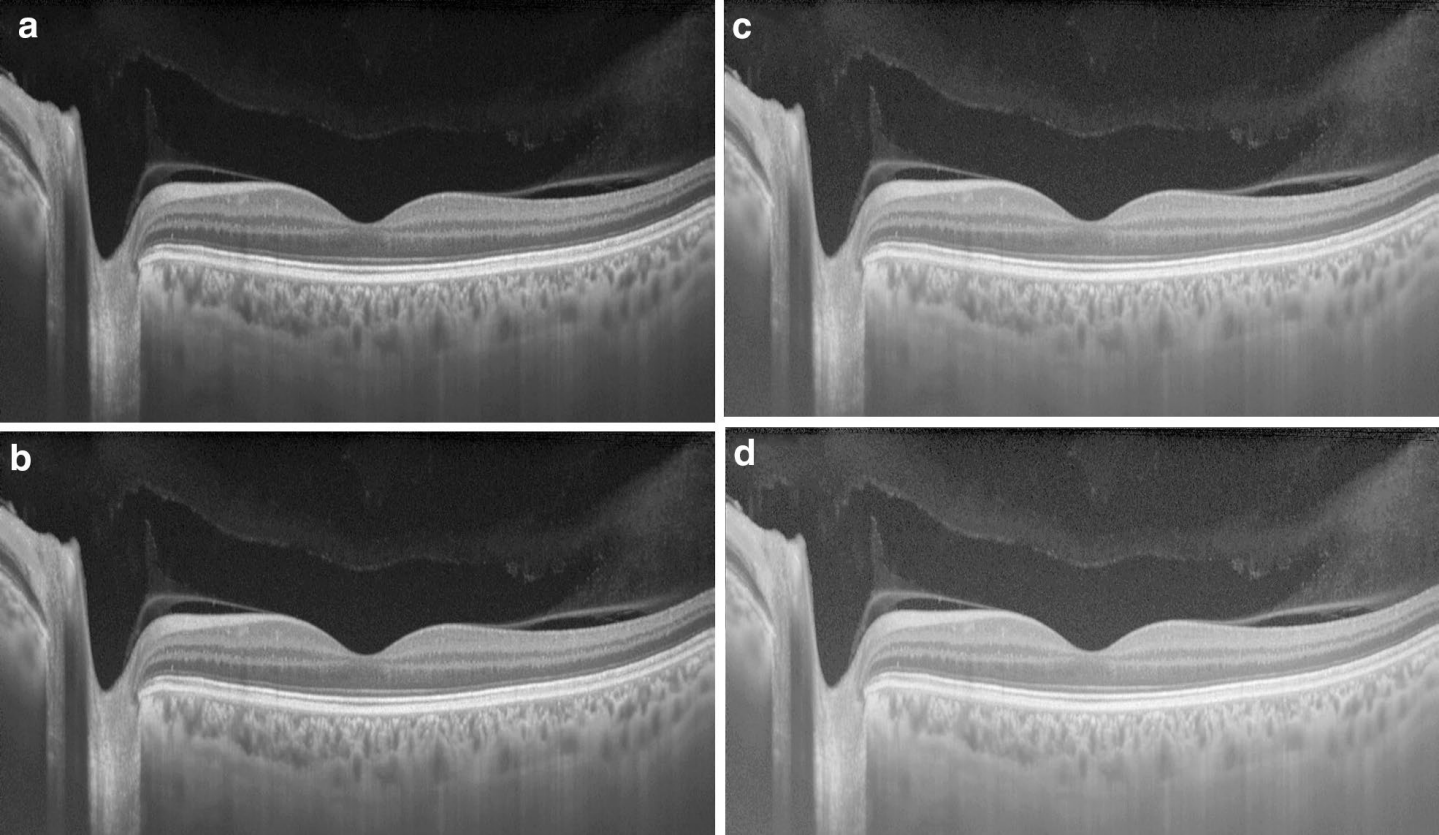
Swept source-OCT high resolution single scan using Enhanced Vitreous Visualization^tm^ (EVV) shows different windows for vitreous visualization, from EVV 0 (**a**), 1 (**b**), 3 (**c**) and 5 (**d**)

SS-OCT has also the enhanced vitreous imaging where the image can be seen in different logarithmic scales, or windows, for better visualization of the vitreous structures [19, 23]. Another complementary approach is to use equalization algorithms to convert the high-dynamic range (HDR) images to a displayable range while preserving contrast, brightness and other fine details [24]. The Triton ^tm^ SS-OCT (Topcon, Tokyo, Japan) uses a feature called EVV (Enhanced Vitreous Visualization^tm^) where these different windows for vitreous visualization can be appreciated (Fig. 2). Structures such as bursa premacularis, or the posterior precortical vitreous pockets (PPVP), the Maregiani area (Clocquet’s canal), posterior cortical vitreous, posterior hyaloid and vitreous opacities of different etiologies are more properly viewed with SS-OCT [20, 25].

The presence of PPVP has a key role in various vitreomacular disorders [26, 27]. The PPVP was recently visualized during vitrectomy using intravitreal injection of triamcinolone acetonide [28]. SD-OCT first allowed in vivo, non-invasive, observation of the PPVP [29]. It was reported that perifoveal vitreous detachment is the primary pathogenic event in idiopathic macular hole formation [30, 31]. In myopic eyes, SS-OCT clearly showed the PPVP increased area [19]. Retinoschisis at the fovea is a common feature in eyes with posterior staphyloma and it appears to be a consequence of vitreoretinal tractions [32, 33]. The SS-OCT was also reported to detect peripheral lesions using a wide field of view that included retinoschisis, diabetic retinopathy, choroidal melanoma and choroidal nevi [34].

Other vitreoretinal interface diseases such as epiretinal membrane and macular pucker, vitreomacular traction syndrome and tractional retinal detachment in proliferative diabetic retinopathy, that are well described with SD-OCT, may also be documented with higher resolution and on broader scans with SS-OCT.

## Choroidal imaging and quantitative evaluation

The choroid receives approximately 70 % of the blood flow in the eye; it has the highest blood flow per unit weight of any tissue in the body, about 20–30 times greater than that of the retina [35–37]. Choroid supplies oxygen and metabolites to the outer retina, retinal pigment epithelium (RPE), the avascular fovea and possibly the prelaminar portion of the optic nerve [38] and serves as a heat sink [39]. The ophthalmic artery, branches to form the central retinal artery and the posterior ciliary arteries (PCAs), the PCAs divide in braches which the two major ones (anterior PCAs) supply the anterior uvea and the smaller short PCAs, usually 20 in number, enter the eye, particularly around the optic nerve and macular region [40]. Venous drainage from the choriocapillaris is mainly through the vortex veins and a minor system of anterior ciliary veins through the ciliary body [35].

The anatomy of the choroid is traditionally described in layers: outer large vessels containing Haller layer, the middle vessels containing Sattler layer and the inner choriocapillaris [35, 41]. The vitality of the choriocapillaris, including the presence of fenestrations, is maintained in part by consecutive secretion of vascular endothelial growth factor (VEGF) by the RPE [42].

OCT techniques that better visualize the choroid is the EDI of the SD-OCT, imaging averaging and SS-OCT. In SD-OCT, depth information is encoded as different frequencies of the interference spectrum, with increasing depth into tissue, echoes beyond the point of detection are known as the “zero delay line.” Spectral domain detection cannot distinguish between positive and negative echo delays, if a retinal structure crosses beyond the zero delay line, it will appear, as a mirror-like image with reversed depth sensitivity. This feature is used to enhance choroidal imaging. This technique was first reported by Spaide and associates as enhanced depth imaging (EDI). EDI is an acquisition software that automatically captures the cross-sectional image with the choroid close to the zero delay line to maximize the sensitivity on the outer limit of the choroid [43, 44].

The SS-OCT has a longer wavelength capable of penetrating tissue more than the SD-OCT, thus both vitreous and choroid can be imaged simultaneously. The first study to measure choroidal thickness (CT) by Margolis and Spaide investigated 54 normal, non-myopic eyes with the EDI of the SD-OCT and found a sub-foveal thickness of 287 µm [45]. Regatieri and associates studied the different layers of the choroid and found a mean CT of 256.8 ± 75.8 μm, mean thickness of the large choroidal vessel layer was 204.3 ± 65.9 μm, and that of the medium choroidal vessel layer–choriocapillaris layer was 52.9 ± 20.6 μm beneath the fovea [46]. The CT seems to decrease with age, however there are patients that present a pronounced loss of CT over time, a new condition named age-related choroidal atrophy [47].

Evaluation of CT is an important diagnostic hallmark of several retinal diseases. Choroidal hyperpermeability is the main feature in CSC [48], CT is increased in those patients [49, 50]. Wood and associates analyzed macular CT with SS-OCT in early AMD in different portions of the posterior pole and there was no significant difference from controls. Switzer and associates found that AMD features were associated with CT thinning [51]. The CT was greater in patients with polypoidal choroidal vasculopathy (PCV) [52, 53]. CT was found important to evaluate in inflammatory diseases involving the posterior segment: Vogh–Koyanagi–Harada [54], multifocal choroiditis and panuveitis, birdshot choroidoretinopathy, posterior scleritis, serpiginous choroidopathy, sarcoidosis and infectious etiologies such as toxoplasmic retinochoroiditis. Choroidal evaluation, both quantitative and morphological may have utility in high myopia, tumors, retina dystrophies and in glaucoma evaluation [43, 55].

CT may also play a role in diabetic retinopathy. Regatieri and associates evaluated 49 eyes of type 2 diabetic retinopathy patients with SD-OCT, the sub-foveal CT was thinner in eyes with diabetic macular edema, or eyes with proliferative retinopathy and previous pan-photocoagulation [56]. A study comparing CT in patients with microalbuminuria and in controls showed a significant thinning in the second group (284.03 ± 12.56 vs. 228.87 ± 14.44 μm; *P* = 0.001; 95 % CI 21.88–88.44) [57].

**Fig. 3:**
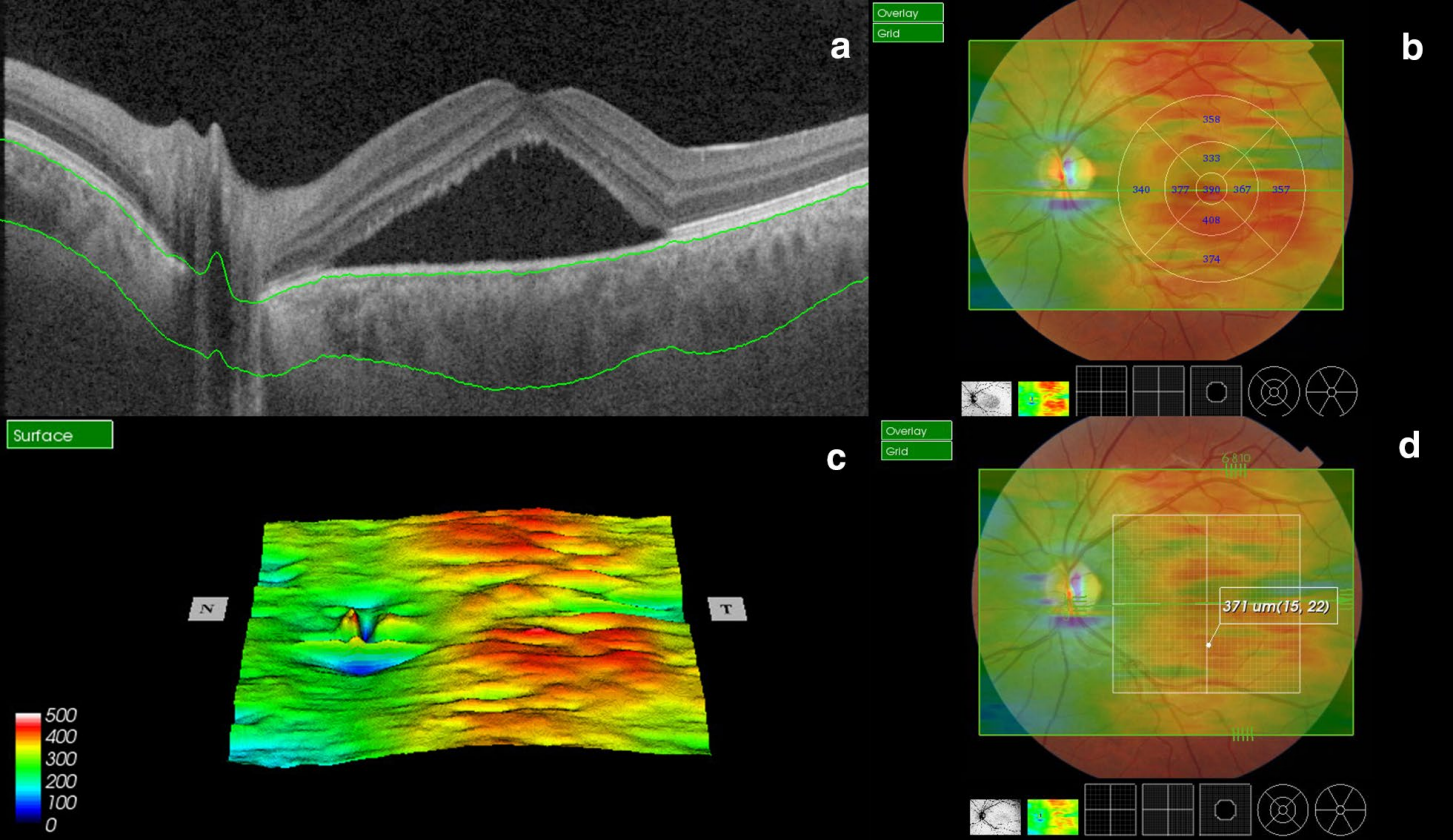
Swept source-OCT single scan shows automatic segmentation layers (**a**) with ETDRS thickness maps (**b**), 3D reconstruction (**c**) and point-by-point automatic measurement of choroidal thickness (**d**)

There have been several reports of foveal CT evaluation, using SS-OCT [58]. Tan and associates compared the SD-OCT and SS-OCT CT measurements where differences by more than 50 μm (SD-OCT thicker) were found [59]. Adhi and associates compared the CT measured with a prototype SS-OCD with a commercially available SD-OCT with and without EDI, there was no significant difference in the measurements on line scans obtained with both systems [60]. In other study, Adhi and associates used the prototype SS-OCT to evaluate the different choroidal layers, as well as the total CT 379.4 μm (SD ± 75.7 μm), the choriocapillaris and small vessels was 81.3 μm (SD ± 21.2 μm) and the choroidal large vessels layer was 298.1 μm (SD ± 63.7 μm) [61]. The commercially available SS-OCT Triton^tm^ (Topcon, Tokyo, Japan) measures the CT with automated segmentation using a viewer software where each sector can be measured separately (Fig. 3). This feature has a promising role on the sectoral analysis and follow-up of retinal pathologies involving the choroid [62].

## OCT angiography and en face OCT

SS-OCT uses a wavelength-tunable laser and a dual-balanced photo detector instead of a broadband super-luminescent diode, spectrometer and high-speed line-scan camera that are used in SD-OCT. SS-OCT offers higher imaging speeds, higher detection efficiencies, improved imaging range and improved depth with reduced sensitivity roll-off. Those features are a fundamental advantage in comparison with SD-OCT and contributed to the improved image quality, including of the choroid, using SS-OCT [63, 64]. These advantages of SS-OCT make it a very useful diagnostic tool to preform En face OCT and OCTA.

Fluorescein (FA) and indocyanine green (ICGA) angiographies are the gold standard for imaging the retinal vasculature, they are dynamic and provide the evaluation of the transit of the dye as well as the well-known diagnostic hallmarks of leakage and pooling of dye [65, 66]. However, with these modalities small retinal vessels and feeder vessels are often obscured by subsequent hyperfluorescence, and they involve the use of intravenous contrast that can result in serious systemic side effects [67].

**Fig. 4:**
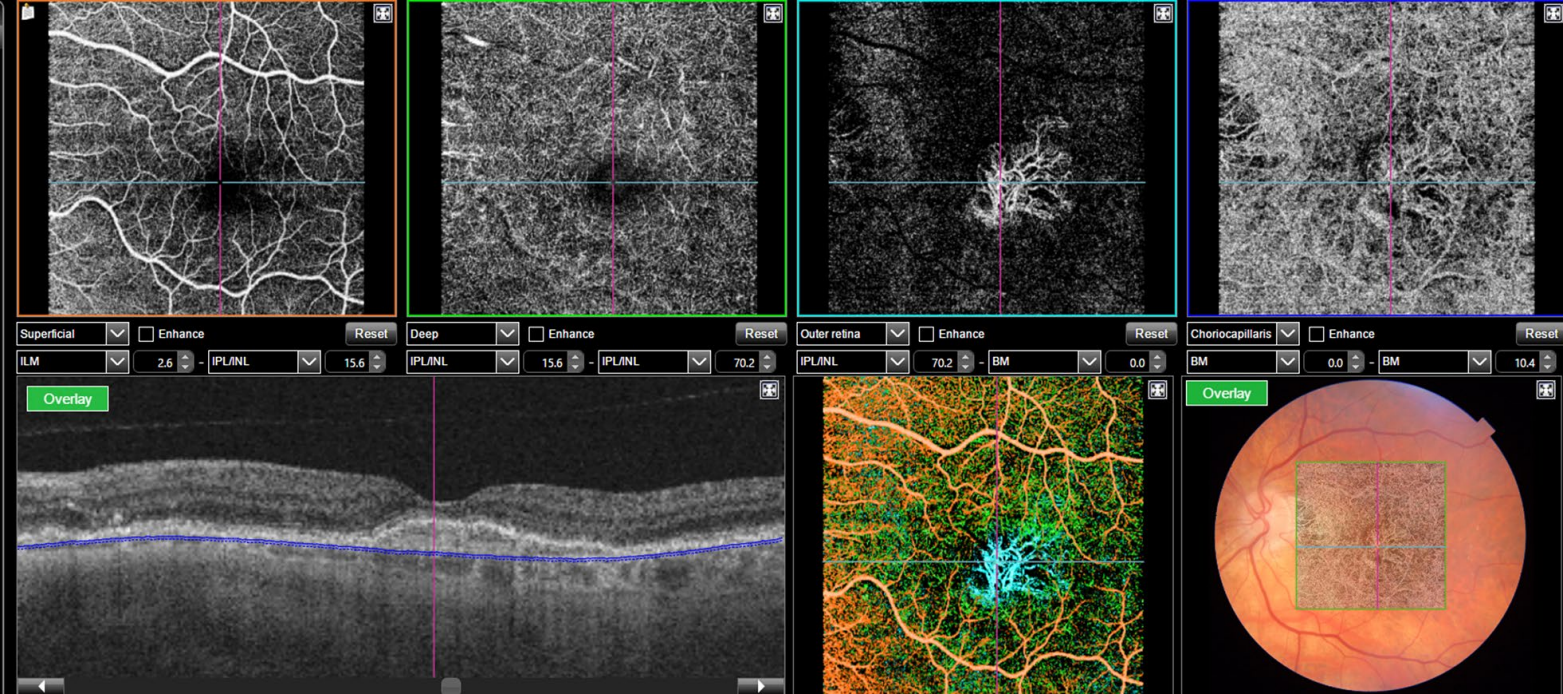
OCT angiography of a patient with exudative age related macular degeneration after treatment with anti-VEGF, however it is still possible to identify choroidal neovascularization bellow the RPE with no signs of inner or subretinal fluid

OCTA is a noninvasive technology that uses the decorrelation motion contrast between rapidly repeated OCT B-scans to visualize blood flow to document the retinal vasculature [68, 69]. OCTA images retinal vessels by detecting variations in the intensity and/or phase properties of the OCT signal over multiple B-scans resulting from the movement of red blood cells [70]. However, there are limitations such as signal attenuation from the RPE, media opacity, and retinal hemorrhage avoiding the full visualization of sub-RPE lesions such as type 1 choroidal neovascularization (CNV). This limitation has been previously reported in SD-OCT and can be overcome with SS-OCT [71, 72] (Fig. 4). OCTA can also miss areas of slow blood flow. It relies on change between consecutive B-scans detecting flow only above a minimum threshold [18].

Novais and associates showed that En face OCTA images from a 1050-nm-wavelength ultrahigh-speed SS-OCT provided a more accurate representation of CNV lesions compared to 840 nm-wavelength SD-OCT [73]. Roisman and associates showed that SS-OCT angiography could identify type 1 neovascularization corresponding to ICGA plaques in asymptomatic eyes with intermediate AMD [74].

The SS-OCT angiography has great potential both for the diagnosis of the three types of CNV and for the follow-up of the anti-vascular endothelial growth factor (anti-VEGF) treatment outcomes [75–77]. The En face SS-OCT may also add useful information for the diagnosis of CNV. Flores-Moreno and associates described that this modality could demonstrate changes in all patients with CNV at different levels of C-scans [78].

**Fig. 5:**
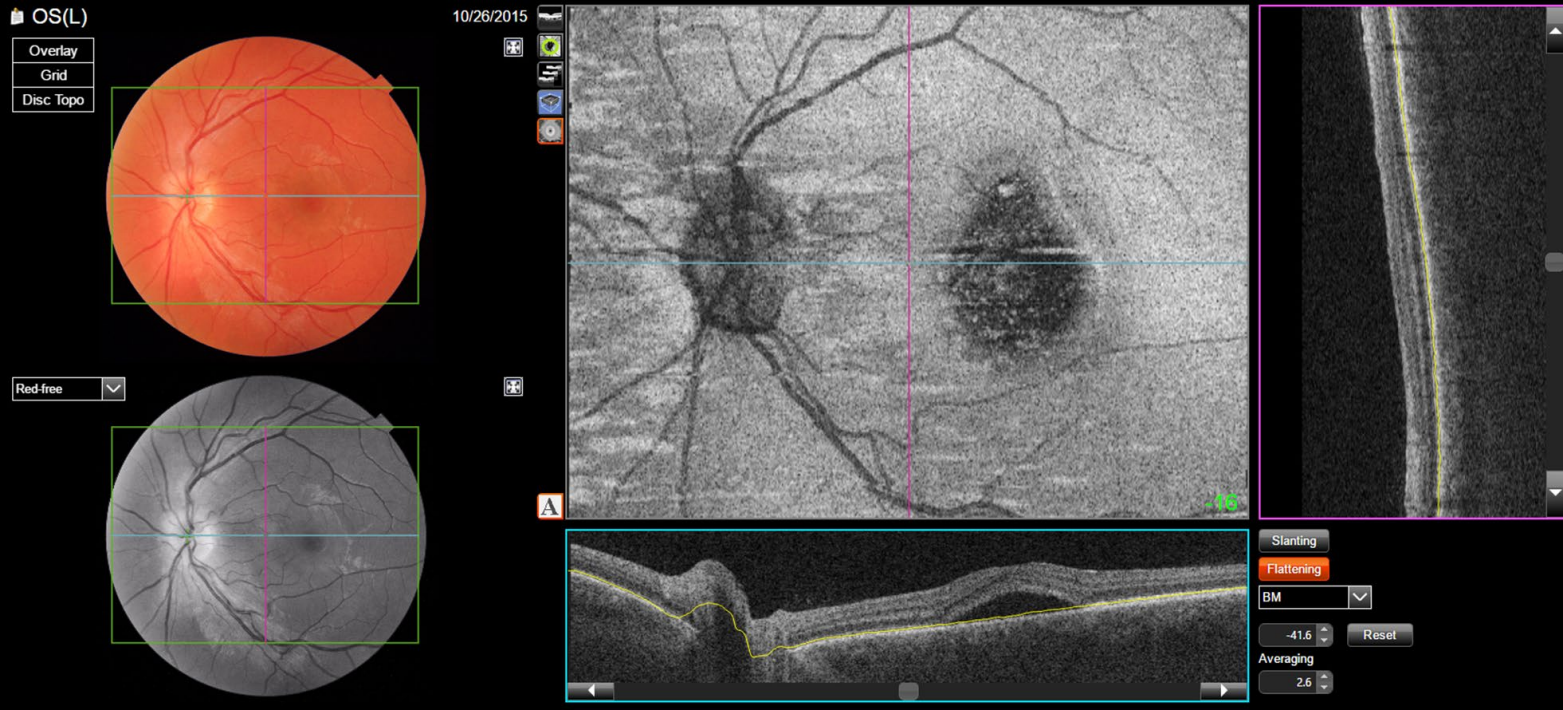
Enface OCT image of a patient with chronic central serous chorioretinopathy at the level of the RPE showing several hypereflective dots under the subretinal fluid and a larger hypereflective spot corresponding to the leaking point on fluorescein angiography

En face SS-OCT images are useful to diagnose pathological features of CSC (Fig. 5). Ferrara and associates showed that at the RPE level there were specific changes in all eyes, which correlated with pathologic changes seen on cross-sectional SS-OCT and other imaging modalities, including fundus auto-fluorescence and angiography. They found in all eyes RPE detachment or loss with correspondent absence of SS-OCT signal at the RPE level. The neurosensory detachment areas showed heterogeneous optically dense dots corresponding to RPE and photoreceptors debris [63]. Bonini Filho and associates demonstrated that OCTA had a sensitivity and specificity comparable with FA to detect CNV in eyes with CSC [79]. The evaluation of the choroid with En face SS-OCT at the level of Sattler’s layer revealed dilation of medium-sized choroidal vessels in two patterns: homogeneous and diffuse dilation of the vessels. The SS-OCT En face images of Haller’s layer revealed diffuse choroidal dilation in the majority of the eyes (80 %) [63].

The evaluation of the choroid with SS-OCT En Face and OCTA may help to understand the pachychoroid spectrum disorders. Densingani and associates evaluated 66 eyes with pachychoroid spectrum and found dilated choroidal vessels in all eyes. Other findings associated with chronic disease: focal choriocapillaris atrophy with inward displacement of deep choroidal vessels, were also appreciated [80].

Another pathology involving the choroid that is better diagnosed with SS-OCT is the polypoidal choroidal vasculopathy (PCV). The diagnosis of PCV has been made based on clinical examination (subretinal orange nodules, haemorrhagic pigment epithelial detachment) and ICGA [81, 82]. OCT has also been described as a useful tool and its features are: “double layer sign” in branching vascular network; notched pigment epithelial detachment (PED) representing the polyp adherent to the undersurface of RPE within a PED; and the polyp itself that looks like a round hyporreflective lumem with a hyperreflective wall adjacent to it [83–85]. SS-OCT is superior in detecting the choroidal-scleral interface and IS/OS lines simultaneously [86]. The use of En face SS-OCT has showed relationship between larger PEDs and small adjoining PEDs that correlated with the polypoidal lesions seen on ICGA [87]. OCTA also improved the multimodal diagnosis of PCV, showing the branching vascular network as well as a round structure with variable reflectivity [88].

The depth of the C-scan is an essential element of the usefulness of the OCTA. The possibility to evaluate different levels such as the superficial vascular plexus, the deep vascular plexus, the choroiocapillaris and the choroidal vessels enhances the comprehension of the different retinal pathologies. The SS-OCT angiography improved the multimodal diagnosis of vascular pathologies of the retina such as macular telangiectasia, diabetic maculopathy, proliferative diabetic retinopathy and occlusive vascular diseases (venous and arterial).

**Fig. 6:**
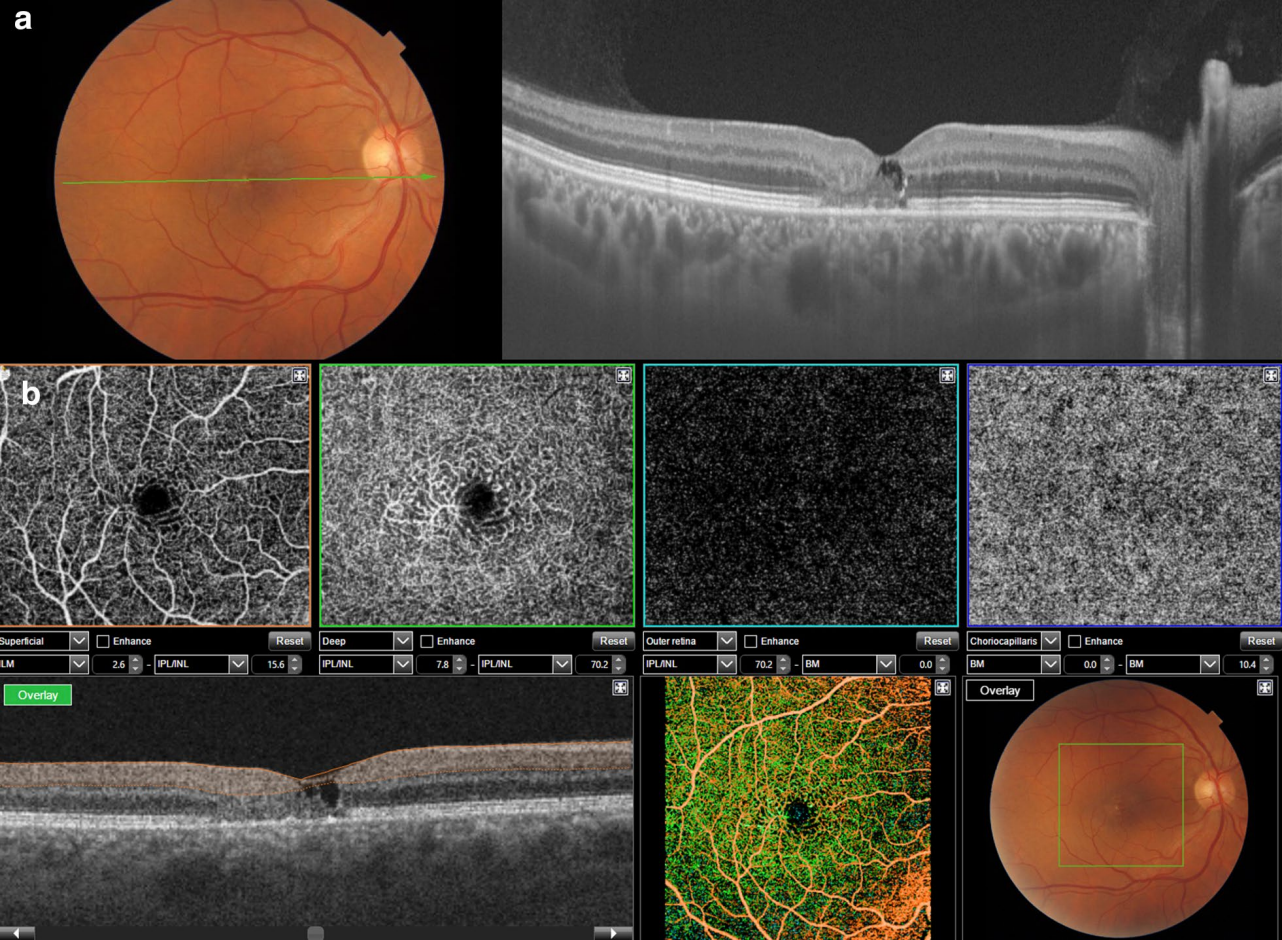
Swept source-OCT high resolution single scan of a patient with macular telangiectasia type 2 with corresponding fundus imaging (**a**), and OCT angiography (**b**) of same patient that shows vascular tortuosity and dilation in superficial and deep layers with no signs of vascular changes on outer retina or choriocapillaris

Macular telangiectasia type 2 (MacTel2) is a disease of the central macula that affects all microvascular layers of the retina and includes neovascularization arising from both the retinal and choroidal circulations. The early changes of MacTel2 are in the temporal aspect of the deep vessels, then into microvasculature around the fovea in superficial vessels and into anastomoses between the deep and the superficial plexus. In some patients, the anastomoses progress to form sub-retinal neovascularization. OCTA reveals these changes at every stage of disease progression [89–91] (Fig. 6).

**Fig. 7:**
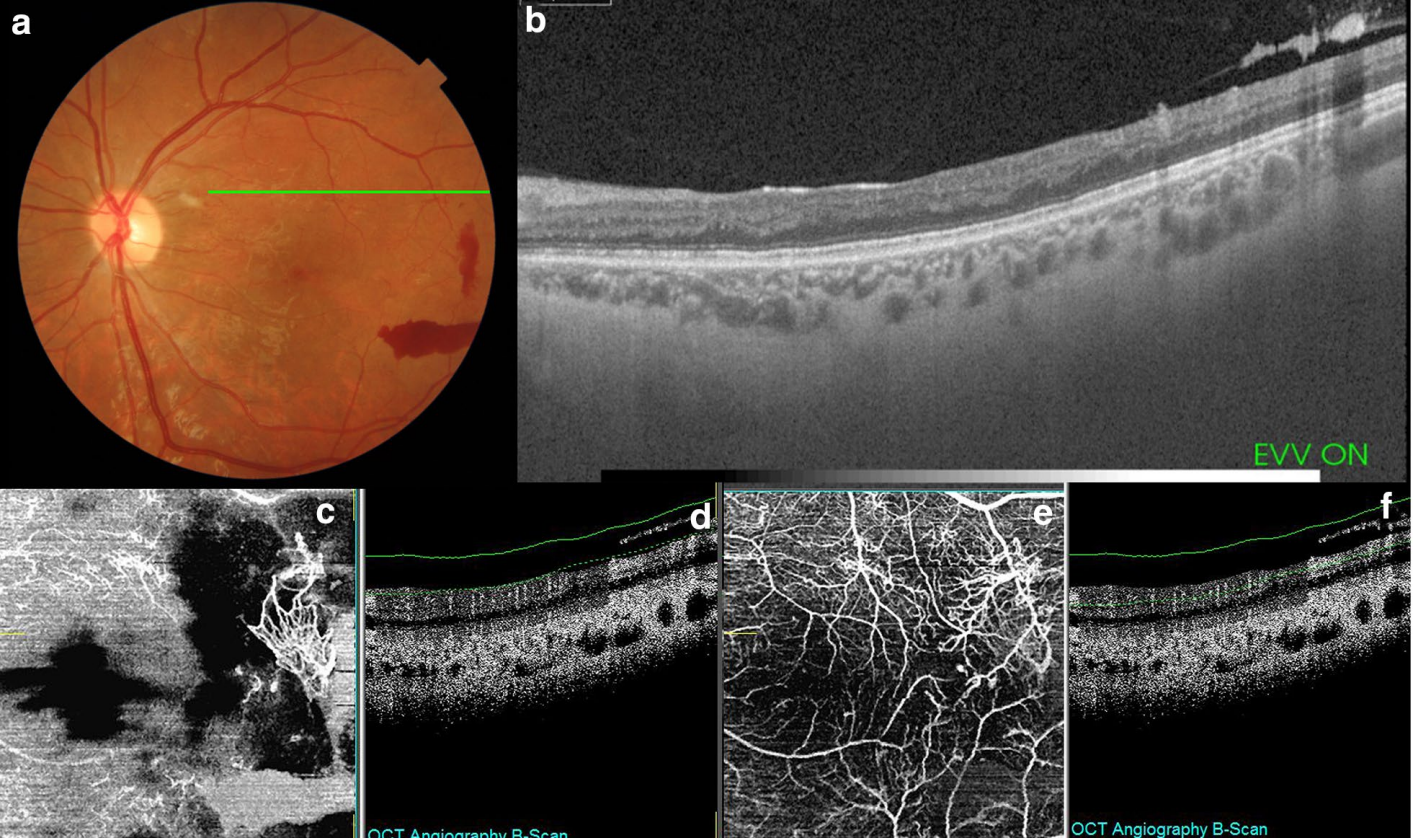
Color fundus imaging of a patient with proliferative diabetic retinopathy (**a**) and single scan swept source OCT image shows partial vitreous detachment with hyperreflective thickening (**b**) that corresponds to retinal neovascularization clearly visible with OCT angiography with vitreous segmentation (**c**, **d**) and superficial segmentation (**e**, **f**)

Diabetic maculopathy and proliferative diabetic retinopathy tomographic features can be appreciated with detail using SS-OCT, En face SS-OCT and OCTA (Fig. 7). Studies demonstrated that OCTA was able to determine relevant vascular changes in subjects with diabetic retinopathy, including microaneurysms, impaired vascular perfusion, some forms of intra-retinal fluid, vascular loops, intra-retinal microvascular abnormalities, neovascularization, and cotton-wool spots. Those findings were largely consistent with FA [92, 93]. Compared with FA, it identified fewer microaneurysms, but located their exact intraretinal depth. OCTA also allowed the precise and reproducible delineation of the foveal non-flow zone [93].

The abnormalities in perfusion seen both in diabetic retinopathy and in retinal vein or artery occlusion are well documented with OCTA. Density maps determining quantitatively this feature were described and have a potential to be an additional tool for the diagnosis or retinal vascular disease. The SS-OCT angiography prototype using variable inter-scan time analysis (VISTA) using a 1050-nm wavelength, 400-kHz A-scan rate is a promising tool to quantitatively determine flow and to assess its impairments in different retinal pathologies [94]. The use of OCTA in retina vein occlusion (RVO) was reported and showed the precise identification of the retinal vasculature as well as the areas of non-perfusion at multiple retinal levels. The limitations of OCTA are a smaller field of view than FA and the absence of dynamic (transit time, speed of flow) and of vascular leakage information [95].

## Summary

The SS-OCT has increased scan speed and a deeper penetration than previous modalities of OCT. The deeper penetration allows the simultaneous detailed documentation of the vitreous and the choroid. It visualizes better the choroidal structures and also sub-RPE pathology such as type 1 CNV. The commercially available SS-OCT has different windows for better vitreous visualization (Enhanced Vitreous View^tm^) as well as segmentation and mapping of choroidal thickness. The En Face OCT and the SS-OCT angiography have improved the diagnosis and the understanding of the features of retinal pathologies including: CNV of different types and etiologies including AMD, CSC and the pachychoroid spectrum, PVC, MacTel, diabetic retinopathy and retinal vascular occlusions. The quantitative evaluation of flow parameters in different pathologies will add important information, especially the ones with vascular etiology.

## Abbreviations

OCT: optic coherence tomography
SS-OCT: swept-source OCT
SD-OCT: spectral-domain OCT
EVV: Enhanced Vitreous Visualization^tm^
AMD: age-related macular degeneration
CSC: central serous chorioretinopathy
MacTel: macular telangiectasia
VMT: vitreomacualar traction
ERM: epiretinal membrane
PPVP: precortical vitreous pockets
PCAs: posterior ciliary arteries
VEGF: vascular endothelial growth factor
CT: choroidal thickness
OCTA: optical coherence tomography angiography
ICGA: indocyanine green angiography
RVO: retina vein occlusion
PCV: polypoidal choroidal vasculopaty
